# Multiscale frameworks for exploring protein energy landscapes: advances in theory and simulation

**DOI:** 10.1007/s10867-026-09718-9

**Published:** 2026-06-26

**Authors:** Patryk Adam Wesołowski

**Affiliations:** Yusuf Hamied Department of Chemistry, University of Cambridge, Lensfield Road, Cambridge, CB2 1EW UK

**Keywords:** Proteins, Potential energy surfaces, Energy landscapes, Multiscale frameworks

## Abstract

Energy landscape theory provides a unifying framework for describing protein structure, dynamics, and function across hierarchies of spatial and temporal scales. In practice, however, protein energy landscapes are never accessed directly; they are represented through a hierarchy of theoretical models, from quantum mechanical potential energy surfaces to coarse-grained potentials of mean force. Each level of description entails a systematic reduction of degrees of freedom and a corresponding transformation of the underlying landscape. In this review, we examine how protein energy landscape representations change under successive approximations to the molecular Hamiltonian, with particular emphasis on the emergence, interpretation, and robustness of landscape concepts across scales. We argue that many simplified models succeed not because they reproduce microscopic interactions in detail, but because key topological features of the landscape, such as funnels, barriers, and competing basins, are preserved under projection. This article also clarifies the physical principles underlying coarse-graining, solvent modelling, and multilevel simulation strategies and demonstrates why energy landscape theory remains predictive despite reduced chemical resolution, highlighting its role as a unifying framework for exploring biomolecular space.

## Introduction

Proteins constitute over half of the dry mass of a typical cell and are responsible for nearly all biological function, including catalysis, signalling, transport, and structural organisation [1]. Despite the extraordinary diversity of their functions, all proteins share a common molecular architecture as polymers of amino acids linked by peptide bonds, with their chemical and physical properties determined by the sequence and nature of the constituent side chains. Interactions between these side chains, together with hydrogen bonding along the protein backbone, give rise to secondary structural motifs such as $\alpha$-helices and $\beta$-sheets, which assemble into domains and ultimately define the tertiary structure of the protein. For a protein to perform its biological role, it must first fold into a specific three-dimensional structure. Under suitable conditions, this native state is uniquely determined by the amino acid sequence, as established by Anfinsen’s thermodynamic hypothesis [2]. The folding process, however, involves collective motions across an enormous number of degrees of freedom, giving rise to a highly complex underlying potential energy surface (PES) [3, 4]. This surface supports a vast number of stationary points, including local minima corresponding to metastable conformations and saddle points that act as kinetic bottlenecks between them. While the native structure often corresponds to the global minimum of the PES or free energy surface under physiological conditions [5], thermodynamic stability alone is insufficient: the native state must also be kinetically accessible on biologically relevant timescales [6, 7].

Energy landscape theory provides a powerful framework for addressing this complexity. Rather than viewing folding as a single reaction pathway, it emphasises the global organisation of configurational space in terms of basins, barriers, and pathways [8, 9]. Within this picture, protein folding emerges as a directed yet stochastic search on a funnelled landscape, comprising an ensemble of convergent pathways leading to the native state [10]. Competing minima and frustrated regions of the landscape give rise to misfolded intermediates and aggregation-prone states, linking folding, function, and pathology within a unified conceptual framework [11–14]. The necessity of such a landscape-based description is underscored by Levinthal’s observation that exhaustive sampling of all possible protein conformations is impossible on physical timescales [15]. Folding must therefore proceed through biased exploration guided by the organisation of the energy landscape, rather than by random search [16]. This view has been central to modern theories of protein folding and has motivated the development of computational approaches capable of characterising ensembles of pathways, metastable states, and transition networks [17, 18]. Despite its widespread use, the term energy landscape is employed in multiple, and sometimes ambiguous, ways. In different contexts, it may refer to a quantum mechanical PES, a classical force-field energy function, a temperature-dependent free energy surface, or a coarse-grained potential of mean force. These representations are related but not equivalent. Each corresponds to a different theoretical resolution, shaped by the degrees of freedom retained and by approximations introduced to render the problem tractable. In practice, biomolecular simulations necessarily rely on successive reductions of complexity: electronic degrees of freedom are eliminated, solvent molecules may be treated implicitly, and atoms are often grouped into effective interaction sites.

The central premise of this review is that many qualitative features of protein behaviour can be understood by examining how energy landscapes transform under systematic reduction of degrees of freedom. From this perspective, coarse-grained models and multilevel descriptions are not merely pragmatic approximations, but well-defined projections of higher-dimensional landscapes. Their success in describing folding, misfolding, and aggregation can be rationalised in terms of the preservation of key topological features, such as funnels, barriers, and competing basins, rather than by detailed reproduction of microscopic interactions. This viewpoint is particularly relevant for systems where misfolding and aggregation play a central role. Protein aggregation into amyloid fibrils underlies a range of neurodegenerative disorders, including Alzheimer’s, Parkinson’s, and Huntington’s diseases [19–21]. Amyloidogenic peptides such as A$\beta$ exhibit multifunnel energy landscapes, supporting multiple competing pathways. Amyloid fibrils form because of a decrease in the solubility of the monomer, controlled by supersaturation [21–23]. Understanding how such landscapes emerge and how they depend on modelling resolution is essential for interpreting both experimental observations and simulation results.

In the following sections, we develop a multiscale perspective on protein energy landscapes [24]. We begin by outlining the hierarchy of theoretical descriptions, ranging from quantum mechanical models to coarse-grained potentials of mean force. We then introduce the formal foundations of PES and free energy landscapes, together with the methodology of the Cambridge energy landscape framework, before examining how these objects evolve across successive levels of resolution. Within this framework, we illustrate how protein energy landscapes emerge at different levels of description and how their essential features are preserved under systematic reduction of degrees of freedom. To this end, we present representative energy landscapes for several protein systems constructed using a range of interaction potentials and modelling strategies. These include landscapes derived from molecular dynamics (MD) trajectories using both atomistic and coarse-grained descriptions, including Chemistry at HARvard Molecular Mechanics (CHARMM) [25], Assisted Model Building with Energy Refinement (AMBER) [26], and UNited RESidue (UNRES) [27] (Fig. 2). We further analyse landscapes of bovine pancreatic trypsin inhibitor (BPTI) obtained with the AMBER [26] and UNRES force fields, as well as energetic profiles along BPTI folding pathway evaluated using subtractive QM/MM scheme of the popular ONIOM (our own N-layered Integrated molecular Orbital and Molecular mechanics) type [28–34] to investigate relative structural stability in the BPTI folding pathway generated with the GFN-FF [35] potential (Fig. 3). Energy landscape representations for amyloid-$\beta$ peptides described using the UNRES [27, 36] potential are also presented (Fig. 4). Particular emphasis is placed on energy landscape methods and multilevel frameworks that link descriptions across scales [24]. Throughout, we focus on how landscape topology governs thermodynamic stability, kinetic accessibility, and model transferability and on why energy landscape theory retains predictive power even as chemical resolution is systematically reduced.

## Hierarchy of theoretical descriptions

### Resolution of molecular models

**Fig. 1:**
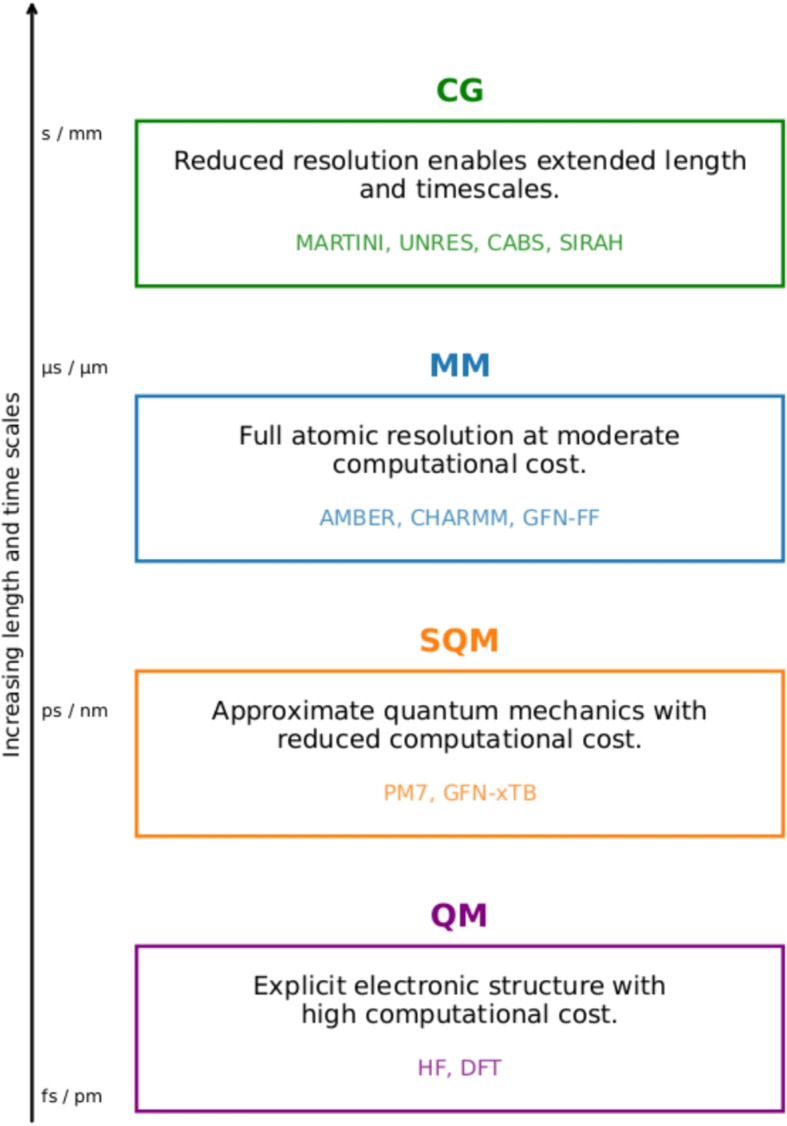
Hierarchy of molecular modelling methods across different resolutions: quantum, semi-empirical, all-atom, and coarse-grained. The plot illustrates the approximate ranges of time scales and system sizes accessible to each method, with example methods indicated. These ranges can be extended by combining multiple resolutions in multiscale modelling approaches

Over the past decade, advances in computational methods, including machine learning approaches such as AlphaFold [37, 38], have enabled remarkable progress in predicting static protein structures. Despite this, predicting folding pathways, along with their associated thermodynamics and kinetics, remains a major challenge due to the inherent complexity of protein energy landscapes, which feature metastable intermediates and structured transition pathways [7, 11, 12, 17, 18, 39]. In Fig. 1, different potentials are presented within the approximate ranges of time scales and system sizes.

Quantum mechanical (QM) methods, such as Hartree–Fock (HF) [40] and density functional theory (DFT) [41, 42], provide the most detailed description by explicitly treating electronic interactions and generating PES for nuclear motion. Within DFT, the systematic improvement of accuracy is commonly rationalised by Perdew’s “Jacob’s Ladder,” which orders exchange–correlation functionals from local and semilocal approximations to hybrid, range-separated, and double-hybrid forms [41, 42]. Among widely used functionals, high-rung range-separated hybrids such as $\omega$B97X-V currently represent the most accurate general-purpose DFT approaches in benchmark studies [43], while meta-GGA methods such as $r^2$SCAN offer a favourable compromise between accuracy and computational efficiency at lower rungs of the ladder [44, 45]. These approaches are essential for accurately describing small molecules, reaction centres, or chemically sensitive regions of proteins, often being used as high-level theory calculations in hybrid schemes. Semiempirical quantum mechanical (SQM) methods, such as PM7 [46] and GFN*n*-xTB [47–49], employ parametrised approximations to extend QM-like accuracy to larger systems and longer timescales, bridging electronic-level and atomistic simulations. GFN2-xTB [50] is a tight-binding method optimised for geometries, vibrational frequencies, and noncovalent interactions, incorporating multipole electrostatics and a self-consistent D4 dispersion model [50, 51]. PM7 [46], in contrast, is an NDDO-based method parametrised for thermochemistry, hydrogen bonding, and reaction energetics. Benchmark studies indicate that GFN2-xTB generally outperforms PM7 in conformational sampling and noncovalent interactions, whereas PM7 remains efficient for small-molecule thermochemistry and covalent bonding applications [51].

All-atom molecular mechanics (MM) force fields, including CHARMM [25], AMBER [26], and GFN-FF [35], treat atoms as point particles interacting through bonded and nonbonded potentials. By integrating out electronic degrees of freedom, these models define smoother effective landscapes while retaining chemically meaningful features such as bond connectivity, sterics, and electrostatics. They allow for the efficient study of folding for small proteins below the millisecond time scale [52, 53]. AMBER protein force fields have evolved considerably, progressively improving backbone and side-chain torsions. The ff99SB [54] introduced corrections to side-chain torsions based on gas-phase QM data. ff14SB [55] refined backbone $\phi /\psi$ torsions using NMR data, enhancing folded-protein stability, while ff19SB [56] incorporates amino-acid-specific CMAP corrections derived from solution-phase QM calculations, improving helical propensities, Ramachandran distributions, and side-chain conformations.

Solvent further shapes the landscape [57]. Explicit models, such as the transferable intermolecular potential (TIP) [58–60] and simple point charge/extended (SPC/E) water models [61, 62], capture detailed solute–solvent interactions, hydrophobic effects, and electrostatic screening at high computational cost [58, 62, 63]. Implicit approaches, including the generalised Born (GB) [64] and Poisson-Boltzmann (PB) models [65], integrate out solvent degrees of freedom, producing effective landscapes with smoothed basin depths and barrier heights. In this sense, explicit solvent represents a high-dimensional, rugged surface close to reality, while implicit solvent acts as a projection that averages fast fluctuations and accelerates sampling. Collectively, these developments provide a hierarchy of options balancing computational efficiency and conformational accuracy. Coarse-grained (CG) models [66], such as UNRES [27], CABS [67], SIRAH [68], and MARTINI [69], group atoms into pseudoatoms or united residues, producing lower-dimensional, smoother landscapes with broader basins and renormalised barriers. Coarse-graining extends accessible timescales into the millisecond–second regime while preserving essential topological features of the energy landscape, including metastable states and dominant transition pathways, thereby enabling studies of protein folding, large-scale conformational transitions, and biomolecular assemblies [36, 66, 70]. As the UNRES model is employed in several energy landscape analyses throughout this review, its key features are summarised here, together with the technical aspects of constructing coarse-grained potentials of mean force (PMF) as an explicit example of how energy landscapes are projected onto reduced, coarse-grained representations.

### UNRES force field

UNRES occupies a distinctive position among coarse-grained protein models by operating at the residue level while retaining chemically meaningful interaction sites. This balance between simplification and physical fidelity enables efficient exploration of protein conformational space over extended timescales, as demonstrated in CASP assessments [71, 72] and a broad range of applications [73, 74]. The model has been successfully applied to systems spanning small globular proteins through to large macromolecular assemblies [71, 72, 75, 76], and ongoing methodological developments continue to extend its scope and accuracy [77, 78]. More recently, UNRES has been integrated into the Cambridge energy landscape framework [21, 36, 79], enabling direct comparison of coarse-grained and atomistic landscapes within a unified framework. The UNRES force field is formulated in terms of restricted free energies, or PMF, which incorporate multibody interactions and explicit temperature dependence from statistical mechanical averaging [80]. In coarse-grained modelling, the PMF plays a role analogous to the Born–Oppenheimer approximation in electronic structure theory: just as electronic degrees of freedom are averaged out to yield an effective potential governing nuclear motion, unresolved atomic and solvent degrees of freedom are integrated out to define an effective landscape for the coarse-grained variables [80]. Hierarchical decomposition of the PMF into Kubo cluster cumulants [81] yields effective energy terms by systematically accounting for correlations between interacting components.

In the UNRES representation, each residue is mapped onto a C$^\alpha$ site with two interaction centres: a united side chain, $\textrm{SC}_j$, and a united peptide group, $p_j$. Chain geometry is defined by virtual bonds, bond angles $\theta _j$, backbone dihedrals $\gamma _j$, and side-chain orientation angles $(\alpha _{\textrm{SC}j},\beta _{\textrm{SC}j})$. The total UNRES energy is expressed as a compact sum [82]:

1 $$\begin{aligned} U =\;&w_{\textrm{SC}} \sum _{i<j} U_{\textrm{SC}_i\textrm{SC}_j} + w_{\textrm{SCp}} \sum _{i \ne j} U_{\textrm{SC}_i p_j} + w_{\textrm{pp}}^{\textrm{VDW}} \sum _{i<j-1} U_{p_i p_j}^{\textrm{VDW}} \\&+ w_{\textrm{pp}}^{\textrm{el}} f_2(T) \sum _{i<j-1} U_{p_i p_j}^{\textrm{el}} + w_{\textrm{tor}} f_2(T) \sum _i U_{\textrm{tor}}(\gamma _i, \theta _i, \theta _{i+1}) \\&+ w_{\textrm{b}} \sum _i U_{\textrm{b}}(\theta _i) + w_{\textrm{rot}} \sum _i U_{\textrm{rot}}(\theta _i, \alpha _{\textrm{SC}_i}, \beta _{\textrm{SC}_i}) \\&+ w_{\textrm{bond}} \sum _i U_{\textrm{bond}}(d_i) + w_{\textrm{ssbond}} \sum _{n_{\textrm{ss}}} U_{\textrm{ssbond}}(d_{\textrm{ss}}) \\&+ w_{\textrm{corr}}^{(3)} f_3(T)\, U_{\textrm{corr}}^{(3)} + w_{\textrm{turn}}^{(3)} f_3(T)\, U_{\textrm{turn}}^{(3)} \\&+ w_{\textrm{dyn}}\, U_{\mathrm {dyn\_ss}} + w_{\textrm{sss}}\, U_{\textrm{sss}} . \end{aligned}$$

Here, $U_{\textrm{bond}}$ describes virtual bond deformations, $U_b$ accounts for bond angle bending, $U_{\textrm{tor}}$ represents backbone torsions, and $U_{\textrm{rot}}$ governs side-chain rotamer preferences. The terms $U_{\textrm{corr}}^{(3)}$ and $U_{\textrm{turn}}^{(3)}$ capture third-order multibody correlations. Each energy contribution is multiplied by a corresponding weight, $w_x$, determined during force-field calibration. The temperature dependence of the leading terms in the cumulant expansion of successive effective energy components is incorporated through scaling functions $f_n(T)$, which modulate the magnitude of each contribution relative to a reference temperature $T_0 = 300\,\textrm{K}$ [81, 83, 84]. Dynamic disulphide bonds are treated using a bimodal switching potential, $U_{\mathrm {dyn\_ss}}$, together with a short-range three-body penalty, $U_{\textrm{sss}}$, which suppresses unphysical trisulphide formation [85]. Disulphides are defined via distances between cysteine side-chain centroids, avoiding the need for additional atoms to represent oxidised or reduced states. This approach overcomes a limitation of all-atom force fields, in which disulphide formation alters molecular connectivity and requires a fixed bonding topology, restricting simulations to native disulphides [33, 34]. In contrast, UNRES allows exploration of alternative disulphide connectivities and interconverting minima within a single, continuous energy landscape. More generally, this illustrates how a carefully constructed coarse-grained potential can preserve key features of the atomistic landscape while enabling enhanced sampling of processes that are otherwise challenging to access with fully atomistic models.

### Hybrid approaches

A central challenge in computational modelling is balancing accuracy and efficiency. QM methods provide unmatched detail but are computationally expensive for large systems, whereas classical force fields and coarse-grained models enable large-scale simulations at the cost of reduced precision for microscopic interactions which might be crucial for some reaction pathways’ energetics. Hybrid and multiscale approaches address this trade-off by combining levels of theory within a single framework. QM/MM schemes partition the system into a chemically critical high layer treated with QM methods and a complementary low layer treated with MM methods [86–89]. Recent extensions introduce intermediate SQM layers, improving electronic accuracy in key regions while maintaining computational efficiency [48, 51, 90]. Such hybrid potentials provide a practical route to model complex biomolecular rearrangements, folding pathways, and energetics with controlled accuracy. One of such schemes, a multicentre *n*-layer ONIOM (MC-ONIOM) scheme [32, 33], is employed here to analyse structures sampled from the BPTI folding pathway. The MC-ONIOM approach [33] was implemented in a standalone Fortran library called lwONIOM, which is freely available under the LGPL-3.0 licence from GitHub [91] and implemented in the CREST programme [34, 92]. Each layer corresponds to a distinct level of theoretical accuracy, with the lowest layer treating the entire protein at a classical force-field level using GFN-FF [35], the intermediate layer comprising three multicentre fragments treated with SQM GFN2-xTB [47], and the highest layer including only the three Cys-Cys disulphide bridges evaluated with QM r$^2$SCAN-3c [45, 93] method (Fig. 3a). More details about the scheme setup are available in [33]. This framework allows the high-level description of chemically critical regions, such as disulphide bridges, to be embedded within a larger protein structure described at a lower level, while maintaining consistency in energies, forces, and vibrational properties across layers, thus providing an efficient multiscale methodology for exploring folding pathways and localised interactions within complex biomolecular systems [33].

From an energy landscape perspective, all computational approaches can be viewed as successive projections of the high-dimensional electronic PES onto lower-dimensional manifolds. QM methods capture the full electronic landscape; all-atom force fields project onto nuclear coordinates; implicit solvent reshapes the solute landscape; coarse-grained models smooth further to explore long timescales; and hybrid methods combine layers to retain accuracy where it matters most. The success of each approach depends not on exact energetic reproduction but on preserving basin connectivity, state hierarchies, and dominant pathways that govern biomolecular behaviour. This unified view rationalises multiscale modelling and guides the integration of methods for studying proteins and other complex biomolecular systems.

## Biomolecular energy landscapes

### Potential energy surface

At the most fundamental level, the energetics of a biomolecule are governed by the electronic Schrödinger equation. Within the Born–Oppenheimer approximation, the separation of electronic and nuclear motion allows the total wavefunction to be expressed as a product of electronic and nuclear components. Solving the electronic problem for fixed nuclear coordinates defines a PES that determines nuclear motion. This surface is a high-dimensional object, defined over the 3*N* nuclear degrees of freedom for a system of *N* atoms. In principle, it encodes all equilibrium and dynamical properties of the biomolecule: local minima correspond to stable or metastable structures, while saddle points define transition states and the kinetic barriers separating them. For realistic biomolecular systems, however, the PES is prohibitively complex. Its dimensionality is enormous, and evaluating it at accurate levels of electronic structure theory is computationally infeasible for systems of biological size. As a result, the PES functions primarily as a conceptual reference rather than a directly accessible object, providing a framework for reasoning about molecular conformations, reaction pathways, and kinetic processes.

While the PES provides a deterministic description of nuclear energetics, biomolecular behaviour at finite temperature is governed by free energy rather than potential energy alone. Thermal fluctuations introduce entropic contributions that are particularly significant in systems with many soft degrees of freedom. Free energy landscapes arise naturally within statistical mechanics through Boltzmann weighting of configurations. Even when all atomic degrees of freedom are retained, the appropriate object for describing equilibrium populations and transition probabilities is not the PES itself, but the free energy surface associated with the canonical ensemble [94]. This distinction is not merely semantic: free energy landscapes are typically smoother than their corresponding PES, as thermal averaging effectively integrates out high-frequency vibrational modes. Consequently, barriers may be reduced, shallow minima may be eliminated, and broad basins may dominate the thermodynamics. The effective landscape at physiological temperature can therefore differ qualitatively from the zero-temperature PES. Importantly, the transition from potential energy to free energy already constitutes a form of landscape projection. Even in the absence of explicit coarse-graining, thermal averaging reshapes the surface on which biomolecular processes occur. There is thus no single, resolution-independent energy landscape for a biomolecule. Instead, the landscape depends on which degrees of freedom are treated explicitly and which are averaged over. Electronic motion, fast nuclear vibrations, solvent fluctuations, and internal conformational modes may all be integrated out to varying extents. From this perspective, energy landscapes form a hierarchy linked by systematic reduction of degrees of freedom, with each level corresponding to a different effective Hamiltonian and, consequently, a different landscape. The central challenge is not to identify a single “correct” landscape, but to understand how landscapes at different resolutions are related and which features persist across levels of description. This hierarchical viewpoint provides the conceptual foundation for interpreting successive approximations, from electronic structure theory to classical force fields and coarse-grained models, as controlled transformations of the underlying PES.

### Exploring energy landscapes

The Cambridge energy landscape framework provides a systematic theoretical and computational approach for analysing the structure, thermodynamics, and kinetics of complex molecular systems. The PES is represented in terms of stationary points, namely local minima and transition states. In this representation, local minima correspond to stable or metastable molecular conformations, while transition states, defined as first-order saddle points of the PES, determine the kinetic barriers separating these states [95]. The resulting network of minima and transition states, commonly referred to as a kinetic transition network [13, 96, 97], provides a compact yet physically transparent description of molecular behaviour [13, 96, 97]. The Cambridge energy landscape framework is realised through a suite of computational tools, most notably the OPTIM [98] and PATHSAMPLE [99] programmes. OPTIM provides robust algorithms for locating and refining stationary points on the PES, while PATHSAMPLE is a driver for successive OPTIM calculations, manages stationary-point databases, and performs thermodynamic and kinetic analyses. Together, these tools enable systematic exploration of the energy landscape using discrete path sampling (DPS) [100–102], in which connected sequences of local minima and transition states are generated and iteratively refined. Local minima are located via geometry optimisation using the limited-memory BFGS (LBFGS) [103, 104] algorithm as implemented in OPTIM. Putative connections between selected minima are generated using doubly-nudged [105, 106] elastic-band [107–109] (DNEB) calculations in OPTIM, with optimisation of the image chain also performed using LBFGS. Candidate transition states are identified as local maxima along these paths and subsequently refined to genuine first-order saddle points using hybrid eigenvector-following techniques [110–113]. Connectivity between stationary points is then established by following steepest-descent paths from each transition state to the adjacent minima. Because the number of stationary points on the PES is expected to grow exponentially with system size [114–116], exhaustive enumeration is neither feasible nor necessary. Instead, thermodynamic and kinetic criteria are used to bias sampling towards the most relevant regions of the landscape [102], allowing key topological features, such as dominant funnels, rate-limiting barriers, and metastable intermediates, to be identified without requiring complete coverage of configuration space. Additionally, Cambridge energy landscape software allows to run the calculations with several potentials including, among others, GFN-FF, GFN-xTB, CHARMM, AMBER, and UNRES.

The potential energy landscape is defined in a space of extremely high dimensionality, making direct visualisation impractical. A common strategy is to project the landscape onto a small number of collective reaction coordinates, thereby reducing the dimensionality to a tractable form. While such projections can be useful, they inevitably obscure aspects of the underlying complexity; in particular, distinct conformations separated by large energy barriers in the full space may appear artificially close or even indistinguishable in reduced representations [96, 117–121]. Disconnectivity graphs provide an alternative representation that avoids explicit dimensional reduction while retaining the essential high-dimensional structure of the landscape [122–125]. In a disconnectivity graph, the vertical axis represents increasing energy, and each terminal branch corresponds to a local minimum positioned at its potential (or free) energy. The horizontal arrangement of minima reflects kinetic connectivity, such that low-energy minima belonging to the same funnel are placed near the centre of that funnel [122, 123]. A funnel is defined as a set of minima that are connected through pathways whose highest intervening transition state lies below the energy at which the corresponding branches merge. The group of structures leading to a particular minimum is usually in the same superbasin, which is related to the folding funnel concept represented by Leopold et al. [10]. Disconnectivity graphs therefore provide a concise yet informative summary of landscape topology. Systems that self-assemble efficiently are typically characterised by a single dominant funnel [123], whereas glassy or frustrated systems, such as amyloids, exhibit multiple competing funnels associated with numerous amorphous minima separated by substantial barriers [21, 126, 127]. Throughout this review, energy landscapes are visualised using a disconnectivity graph.

### Free energy calculations and enhanced sampling

Molecular dynamics (MD) simulations remain widely used for exploring biomolecular systems, despite well-known challenges associated with inadequate sampling in the presence of large free energy barriers and the resulting breakdown of ergodicity. A variety of enhanced-sampling techniques based on explicit dynamical propagation have been developed to mitigate these limitations and improve access to rare events [128–139]. These approaches aim to improve exploration of configurational space and enable the reconstruction of free energy surfaces along selected collective variables. Among the most widely used methods is umbrella sampling, in which a series of biased simulations is performed to enhance sampling in predefined regions of configuration space. The resulting distributions are subsequently combined, for example, using the weighted histogram analysis method (WHAM) [94, 140], to recover the underlying free energy profile. Closely related approaches include adaptive biasing force methods, which estimate free energy gradients directly, and metadynamics, where a history-dependent bias potential is constructed iteratively to discourage revisiting previously sampled configurations [141, 142]. These methods are particularly effective for exploring rare events and activated processes in complex biomolecular systems. Such techniques provide a complementary perspective to energy landscape approaches based on stationary-point databases. While enhanced sampling methods typically rely on a reduced set of collective variables and therefore involve an explicit projection of the high-dimensional landscape, they yield free energy profiles that are directly relevant to thermodynamic observables and experimentally measurable quantities.

Markov state models (MSMs) [136, 138, 139, 143–146] provide a powerful framework to reconstruct free energy landscapes and long-timescale dynamics from ensembles of molecular dynamics trajectories. Configurational space is discretised into metastable states, typically via clustering in a low-dimensional representation, and transitions between these states are used to capture the system’s kinetics. The stationary distribution of the MSM yields relative free energies of the basins, while the slowest dynamical modes capture dominant conformational transitions and associated timescales. By integrating data from many short or biased trajectories, including those generated with enhanced-sampling techniques, MSM offer a consistent thermodynamic and kinetic description of complex, rugged energy landscapes that would be otherwise inaccessible to straightforward MD simulations. In this sense, MSMs provide a trajectory-based analogue of the kinetic transition networks obtained in the Cambridge framework. However, whereas MSMs are derived from finite-temperature dynamical sampling and depend on the choice of state decomposition and lag time, DPS constructs networks from explicit identification of stationary points and transition states, yielding a description that is independent of dynamical sampling but contingent on the accuracy of the underlying potential energy surface. Kinetic information can be extracted in both the Cambridge energy landscape framework and MSM, but the underlying constructions differ significantly. In the DPS approach, global kinetics are obtained by representing the system as a network of stationary points, where rate constants are computed assuming Markovian dynamics between local minima. This assumption is justified when intrabasin equilibration is fast compared to interbasin transitions, such that memory of prior configurations is effectively lost. MSMs employ a similar Markovian framework, but construct the transition network directly from molecular dynamics trajectories at finite temperature. Configurational space is partitioned into metastable states, and transition probabilities are estimated over a chosen lag time. The resulting kinetics therefore depend on the quality of sampling, the choice of state decomposition, and the timescale separation between intra- and interstate motion.

The key distinction is that DPS derives kinetics from the underlying potential energy surface via explicit identification of minima and transition states, and is therefore not limited by dynamical sampling, while MSMs infer both thermodynamics and kinetics from trajectory data, providing a direct connection to free energy landscapes. As a result, the two approaches are complementary: DPS offers detailed insight into landscape topology and barrier structure, whereas MSMs provide a statistically grounded description of finite-temperature dynamics and long-timescale behaviour [147]. Methods based on geometry optimisation, which calculate the barriers directly, can address any time scale in principle, and aim to explain emergent thermodynamic and kinetic properties in terms of the underlying energy landscape [11, 13, 17], but are computationally expensive for larger biomolecules. Taken together, these approaches illustrate the complementary strategies available for exploring biomolecular energy landscapes. Methods based on MD and enhanced sampling provide direct access to free energy surfaces and finite-temperature dynamics, while geometry-based approaches offer detailed insight into the underlying topography of the potential energy surface.

More recently, disconnectivity-graph concepts originally developed for analysing kinetic transition networks [13, 96, 97] have been adapted to extract additional structural insight directly from MD trajectories with the MDDG programme [148]. In its present formulation, MDDG focuses on describing the conformational space sampled in an individual simulation. This contrasts with MSM, which are constructed to extract equilibrium kinetic information from either long continuous trajectories or collections of shorter simulations [136, 139, 143–146]. General procedures allow configurations sampled during a simulation to be mapped onto an effective energy landscape, enabling identification of the hierarchy of proxy minima and intervening barriers explored on MD timescales. When represented as disconnectivity graphs, these effective landscapes provide a concise and physically meaningful summary of the sampled configurational space, particularly when combined with a suitable order parameter. To suppress high-frequency thermal fluctuations while retaining the organisation of thermally accessible basins, potential energy time series are processed using Savitzky–Golay smoothing [149]. This treatment reveals the underlying landscape structure sampled by the trajectory, with proxy minima that correspond closely to quenched configurations obtained by geometry optimisation. Although the present implementation implicitly exploits the limited sampling of conventional MD by assuming that configurations are not revisited, it nonetheless demonstrates the ability of disconnectivity graphs to recover landscape organisation directly from MD data. Large energy differences between proxy minima should be interpreted as visual proxies for relative ordering and connectivity rather than literal barriers crossed during the simulation. Further methodological details are available at Ref. [148].

## Energy landscapes from MD trajectories

**Fig. 2:**
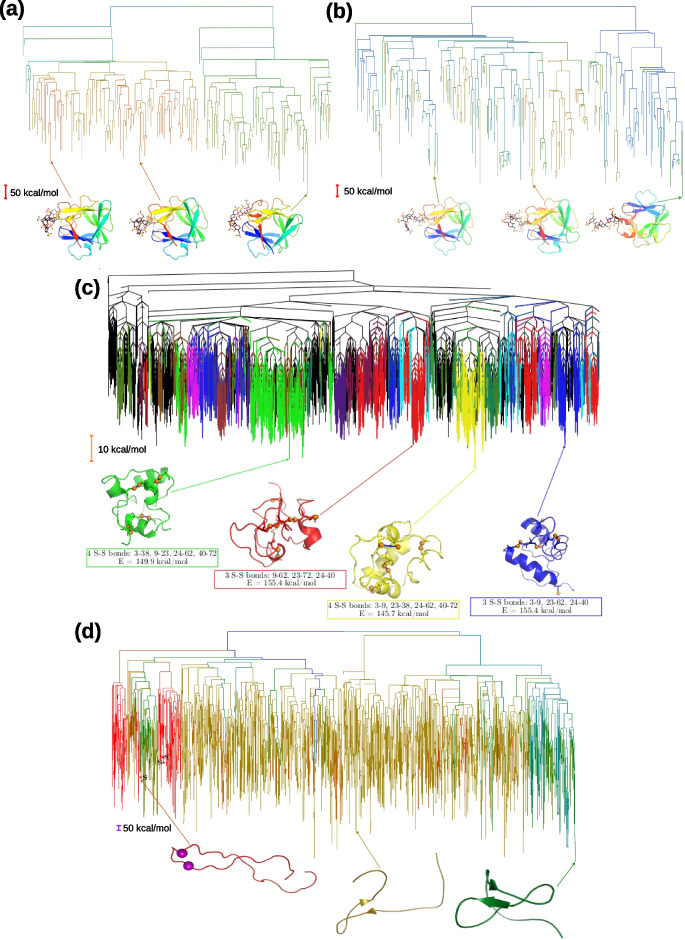
**a**, **b** FGF-2–heparin dp6 complex described using AMBER and CHARMM force fields; branch colour encodes the GAG RMSD, with red indicating the smallest and blue the largest values (17 Å), and arrows marking the lowest-energy minima. The protein is shown as a rainbow cartoon, and the GAG by element (C, dark grey; O, red; N, blue; S, yellow). **c** 1HYP protein described with the UNRES potential, coloured by disulphide-bond topology; the 15 most frequent patterns are shown in colour, with rare patterns in black. Highlighted deep minima were reconstructed using PULCHRA [151] and refined with SCWRL4 [152], with arrows indicating the corresponding proxy minima. **d** A$\beta _{42}$ monomer simulated with CHARMM36m; branch colour represents the number of residues in $\beta$-sheet conformation, with red indicating the smallest and blue the largest values (12). Representative structures with varying $\beta$-sheet content are shown, and the D23–K28 salt bridge in minima labelled S is highlighted as purple spheres. Figures adapted with permission from Ref. [148]

We present energy landscapes for several representative systems described using different interaction potentials. For protein–glycosaminoglycan (protein–GAG) complexes, two landscapes are analysed, constructed using the AMBER and CHARMM. We then present the energy landscape obtained with the UNRES potential incorporating dynamic disulfide bonds, followed by the energy landscape of the A$\beta _{42}$ monomer described using the CHARMM potential (Fig. 2). Energy landscapes derived from molecular dynamics trajectories, the details of which are provided in Ref. [148], were constructed using the MDDG programme. The corresponding disconnectivity graphs were generated using the disconnectionDPS approach [150].

In Fig. 2a, b, the branching structure of the disconnectivity graphs shows a clear correlation with the GAG RMSD, with this relationship being more pronounced for the AMBER description. In this case, the proxy minima cluster at relatively small RMSD values, indicating more localised sampling around the reference configuration. By contrast, the CHARMM minima span a wider RMSD range, reflecting a broader exploration of configuration space. Such behaviour suggests that successive MD frames are more likely to bypass intermediate minima, causing MDDG to infer comparatively higher effective barriers. This difference is consistent with the CHARMM potential energy surface exhibiting shallower minima in the vicinity of the starting configuration along the GAG RMSD coordinate. These observations align with earlier reports highlighting systematic differences between AMBER and CHARMM in their descriptions of protein–GAG energy landscapes and in their treatment of solvent effects [153, 154]. Both force fields are widely applied to protein systems, including protein folding. In particular, AMBER ff14SB and CHARMM36m have been used to simulate folding in systems such as CLN025, villin, and the WW domain, with AMBER favouring helix formation and CHARMM providing more balanced sampling of folded and disordered states [155]. Importantly, even when simulations are projected at the all-atom level, differences between potentials persist, so the choice of force field should be guided by the specific system under study and the properties one aims to investigate.

In Fig. 2c, the influence of disulphide bonding on the explored PES is highlighted by colouring the disconnectivity graph according to canonical S–S connectivities. The 15 most commonly sampled disulphide patterns are shown using distinct colours, while less frequently occurring motifs are grouped and displayed in black. A strong correlation between individual funnels is evident, indicating that disulphide connectivity acts as a primary organising variable of the sampled landscape. Large, uniformly coloured regions correspond to funnels in which the disulphide network is preserved, whereas changes in colour along connecting branches mark transitions over barriers that coincide with disulphide exchange events. Funnels distinguished by different colours therefore represent alternative disulphide arrangements within the same overall basin. The clear separation of these funnels illustrates how dynamic disulphide bonding gives rise to multiple structural hierarchies on a shared energy landscape, providing a direct visual link between S–S rearrangements and landscape organisation. The frequent transitions between distinct connectivities observed among the smoothed minima thus reflect genuine disulphide reshuffling events rather than noise, leading to well-defined basins that are naturally classified by their disulphide topology.

Figure 2d shows the energy landscape of the A$\beta _{42}$ monomer reconstructed from a CHARMM36m MD trajectory. In the original study [156], the authors obtained the free energy surfaces (FESs) of the monomer and dimer from MD simulations using the all-atom CHARMM36m force field, sampling 6 $\mu$s for the monomer and 24 $\mu$s for the dimer. Each frame of the trajectories was characterised using the distribution of reciprocal interatomic distances (DRID) metric [157, 158], a structure-specific dimensionality reduction method known to preserve system kinetics effectively. C$^\alpha$ atoms of key residues (D1, F19, D23, K28, L34, and A42) were used as reference atoms, and the first three moments of their DRID distributions defined the state vectors, resulting in a 3$N \rightarrow$ 3$N_c$ dimensionality reduction, where *N* is the number of atoms and $N_c$ the number of reference atoms. After clustering, the resulting trajectories of states were used to construct a rate matrix of transitions, which was translated into a free energy surface (FES). The resulting FES were visualised using disconnectivity graphs, revealing an inverted, highly disordered funnel with the lowest free energy states corresponding to structurally heterogeneous conformations, consistent with previous studies [156].

For the MDDG analysis (Fig. 2d), only the monomer MD trajectory was used to construct a disconnectivity graph representing the portion of the landscape explored in the single MD simulation. The trajectory exhibits a frustrated organisation, with many disordered proxy minima of comparable energy separated by substantial effective barriers. Colouring the branches by the number of residues in $\beta$-sheet structure (0–12) reveals a clear correlation with branching topology: most minima display low $\beta$-sheet content, indicating that the sampled region is dominated by flexible, disordered states. The apparent barriers are likely overestimated due to sparse temporal sampling (10 ps), which can skip intermediate configurations and inflate barrier heights inferred by MDDG. Consequently, the disconnectivity graph represents only the sampled portion of the landscape rather than the global PES. Within this region, salt-bridge-containing conformations with short D23–K28 distances ($<4\,\mathring{A}$) occur exclusively in disordered funnels, suggesting that although such motifs are relevant to early aggregation [156, 159, 160], the higher $\beta$-sheet-content states explored here do not correspond to the $\beta$-hairpin structures central to A$\beta _{42}$ oligomerisation. Overall, this reinforces the picture of a rugged, frustrated landscape dominated by disorder at the monomer level [156, 159, 160].

We illustrate how the character of protein energy landscapes depends on the choice of potential and the level of coarse-graining, highlighting their hierarchical nature. Protein–GAG complexes are described using both AMBER and CHARMM, with AMBER yielding more localised proxy minima and CHARMM exploring broader configurational space, reflecting differences in barrier heights and landscape roughness [148, 153, 154]. The UNRES potential with dynamic disulphides captures multiple funnels organised by S–S connectivity, showing how covalent rearrangements define subbasins within a coarse-grained landscape. For the A$\beta _{42}$ monomer, CHARMM36m MD sampling produces a disordered, frustrated funnel [156, 161] dominated by low $\beta$-sheet content, where apparent barriers are inflated due to sparse temporal sampling [148, 156, 159, 160]. These examples demonstrate that different potentials emphasise distinct features, local flexibility, covalent connectivity, or disordered states, while the disconnectivity graph framework provides a unifying representation of sampled landscapes across resolutions.

## BPTI energy landscapes

**Fig. 3:**
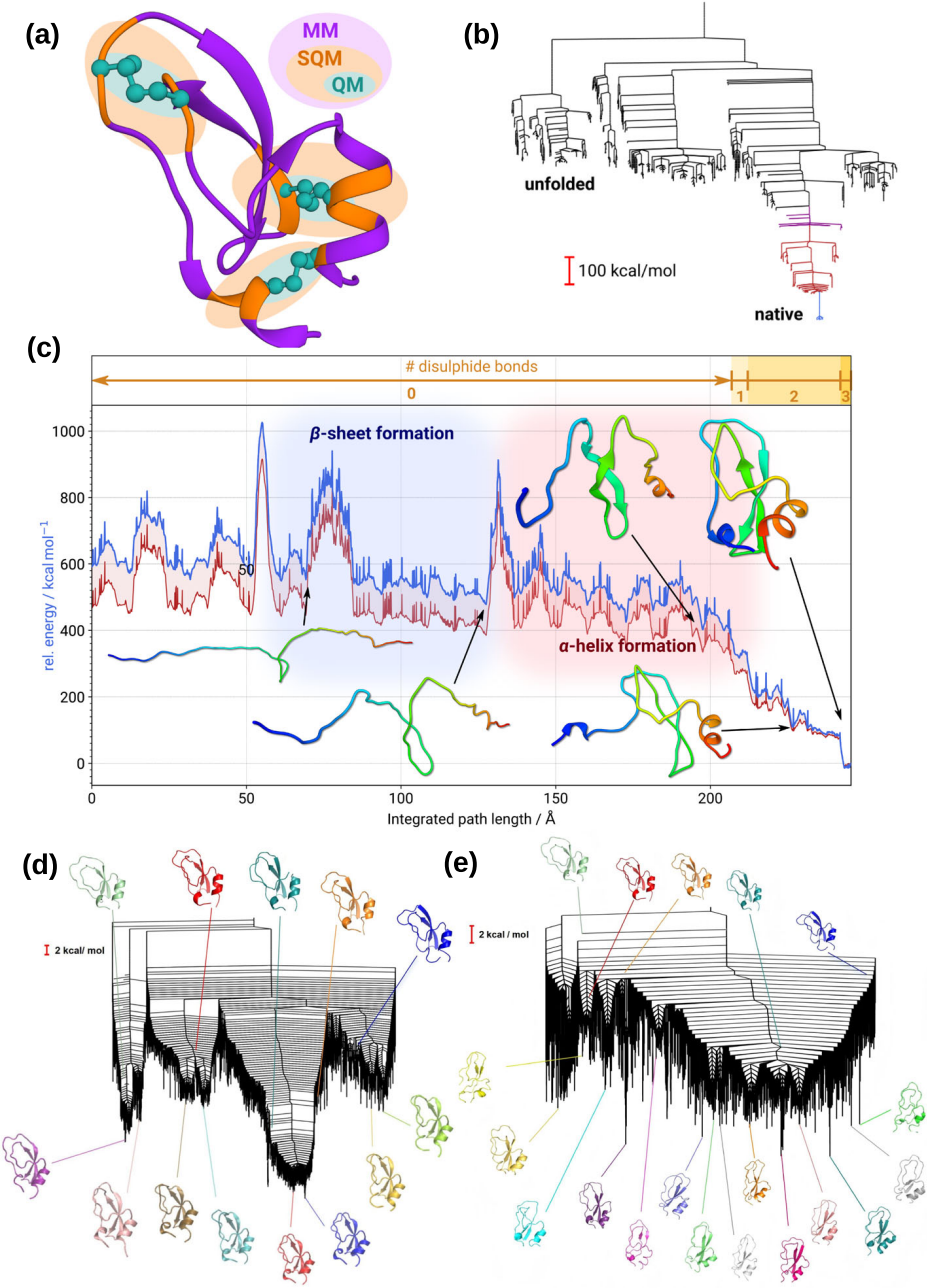
**a** Three-layer, three-centre ONIOM(r$^2$SCAN-3c:GFN2-xTB:GFN-FF) partitioning of BPTI, with each centre built around a Cys–Cys disulphide bond. **b** Disconnectivity graph at the GFN-FF level corresponding to the pathway in **c**. **c** Folding pathway of BPTI showing energy (left) and the number of disulphide bonds along the path (yellow, top). The red curve shows single-point gas-phase energies re-evaluated with ALPB implicit solvation [170]. Regions of $\beta$-sheet and $\alpha$-helix formation are shaded blue and red, respectively. **d** Disconnectivity graph of the BPTI potential energy landscape using the AMBER potential; the four main funnels and selected minima are shown. **e** Disconnectivity graph using the UNRES potential, showing nine principal funnels and representative minima. Figures adapted with permission from [33, 79]

Bovine pancreatic trypsin inhibitor (BPTI) is one of the most extensively studied proteins for analysing folding pathways (Fig. 3a). BPTI is a monomeric, globular polypeptide comprising 58 residues, formed from 16 distinct amino acids, which fold into a stable and compact tertiary structure. Its crystal structure reveals a twisted $\beta$-hairpin, N-terminal and C-terminal $\alpha$-helices, and three disulphide bonds (Cys30–Cys51, Cys5–Cys55, Cys14–Cys38) [162]. The stabilising role of these disulphide bonds in the native state has been extensively characterised [33, 79, 163–166]. The folding pathway of BPTI is energetically favoured towards a structure with a well-defined tertiary arrangement. Interestingly, transient disulphide bonds, such as Cys5–Cys30, Cys5–Cys14, Cys5–Cys38, and Cys5–Cys51, form along the pathway but are absent in the final native state [165]. BPTI’s modest size and well-characterised folding behaviour make it an ideal system for probing the thermodynamic stability of disulphide bonds and their contribution to overall structural stability. In this study, the lwONIOM method within CREST [28–34, 34, 91] was employed to investigate relative structural stability along the BPTI folding pathway. Several previous studies have applied various QM/MM models to BPTI [167–169], with particular focus on the formation of the three native disulphide bonds. Initially, the folding pathway towards the native state was sampled using the all-atom GFN-FF force field [35]. Subsequently, a multiscale approach was employed to calculate free energies for selected points along the folding path: DFT calculations at the r$^2$SCAN-3c level [45, 93] were used to accurately describe disulphide bond stability, neighbouring regions were treated with GFN-xTB, and the remainder of the structure was described with GFN-FF (Fig. 3a).

Before assessing disulphide bond stability via an lwONIOM approach, GFN-FF with the OPTIM programme was employed to map the BPTI folding pathway (Fig. 3c). GFN-FF, a robust dissociative force field [35], enables modelling of homolytic cleavage and reformation of disulphide bonds, which was exploited using the DNEB method starting from the native topology. The resulting pathway, approximately 250 Å in integrated length (Fig. 3c), shows that the most pronounced energetic stabilisation occurs in the final third, coinciding with disulphide bond formation. Secondary structures fold hierarchically, with $\beta$-sheet formation preceding the C-terminal and N-terminal $\alpha$-helices. The disconnectivity graph of the stationary point database (Fig. 3b) highlights distinct funnels corresponding to secondary structure motifs and the main disulphide bond funnel, associated with transition states (Cys5–Cys55), (Cys30–Cys51), and (Cys14–Cys38). Implicit solvation evaluated via ALPB [170] reduces the energy difference between folded and unfolded states, particularly stabilising unfolded conformations due to their larger surface area, while leaving the overall pathway largely unchanged (Fig. 3c). Although the GFN-FF pathway treats disulphide bonds as homolytically cleaved radicals, it provides a useful model for relative stability benchmarking. lwONIOM calculations reveal that oxidative disulphide formation contributes significantly to folding energetics, with reaction energies consistent with experiment [171]. The pathway demonstrates that disulphide bond formation is a major stabilising factor, contributing roughly two-thirds of the energy difference between native and unfolded states. Implicit solvation attenuates noncovalent interactions but does not significantly alter relative disulphide stabilities.

Additionally, we present BPTI energy landscapes obtained with AMBER (Fig. 3d) and UNRES (Fig. 3e). For both potentials, the energy landscape explored contains native-like structures. Calculations employed the Cambridge energy landscape framework, with DPS calculations initiated from five structures extracted from long-timescale MD simulations by Shaw et al. [163]. Despite the different levels of resolution, both models produce broadly similar folded ensembles, strongly constrained by the three disulphide bonds. For AMBER, representative minima are highly compact and closely resemble the crystallographic structure [79]. In contrast, UNRES minima, as expected for a coarse-grained potential, exhibit slightly greater conformational flexibility while still sampling folded, native-like states. The correspondence between minima on the two surfaces is not one-to-one: for example, the global minimum in each potential is not identical [79]. This mismatch reflects intrinsic differences in the potentials and the challenges of mapping all-atom to coarse-grained landscapes, which inherently sample different conformational spaces. Nevertheless, both AMBER and UNRES capture native-like structures close to the experimental configuration, with UNRES exploring a wider range of conformations over comparable computational times due to its reduced resolution. Biologically, this indicates that both force fields sample the region of the landscape surrounding the experimental structure, capturing stable transition states and resulting in similar structural ensembles.

**Fig. 4:**
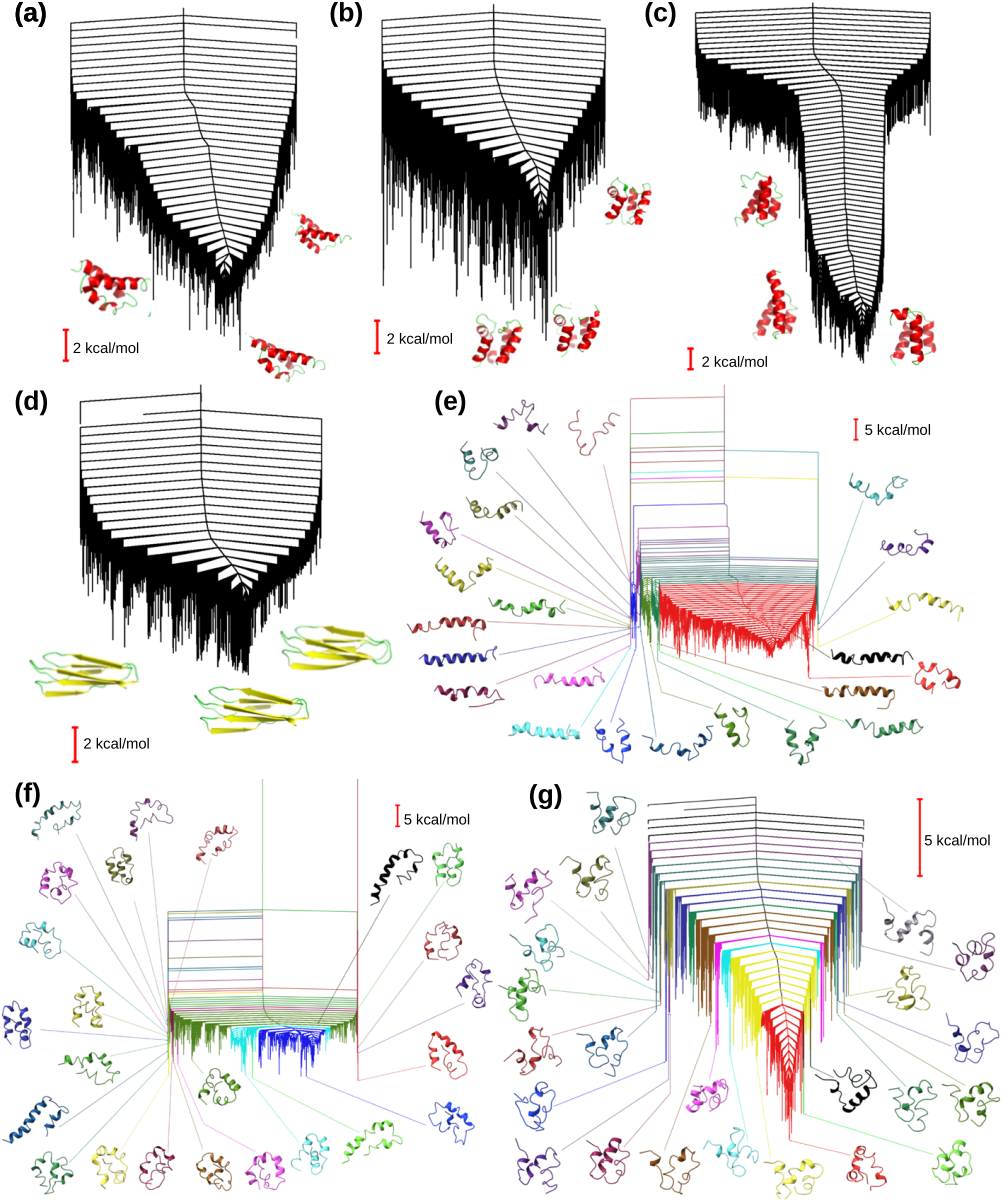
All disconnectivity graphs presented here were obtained using the UNRES potential [36] for different systems: **a** Fc binding protein, **b** apo calbindin D9k protein, **c** de novo protein, **d** Lactococcin 972 protein, **e** A$\beta _{28}$ peptide, **f** A$\beta _{40}$ peptide, and **g** A$\beta _{42}$ peptide. In **e**–**g**, the colours used in the disconnectivity graphs correspond to the coloured structures representative of the basins they represent. Adapted with permission from Refs. [21, 36]

Overall, BPTI provides a compelling case study for how protein folding and stability can be interpreted through the common language of energy landscapes across multiple levels of resolution. From dissociative GFN-FF pathways and lwONIOM free energy refinements to fully atomistic AMBER and coarse-grained UNRES landscapes, all models converge on a picture in which folding is funnelled towards native-like states strongly stabilised by the formation of three disulphide bonds. While the detailed topology of the landscape, such as the ordering of transition states, the depth of funnels, and the correspondence between minima, depends sensitively on the underlying potential and treatment of solvation, the global organisation of the landscape remains robust. Implicit solvent effects modulate relative energetics without qualitatively altering the folding routes, and reduced-resolution models broaden conformational sampling while preserving native-state structures. Together, these results highlight energy landscape theory as a unifying platform for comparing force fields and multiscale models, enabling consistent interpretation of folding mechanisms, stability, and thermodynamics across atomistic, coarse-grained, and hybrid descriptions.

## UNRES energy landscapes

Finally, energy landscapes described by the UNRES potential were explored [36]. We first considered small, well-characterised proteins with clearly defined native structures, initiating landscape explorations from their experimental conformations. For such systems, a single-funnel topology is expected and indeed observed (Fig. 4a–d). Analysis of four representative proteins, each dominated by different secondary structure motifs, provides a geometric characterisation of their energy landscapes and serves as an important benchmark for assessing the coarse-grained UNRES potential in comparison with the all-atom AMBER force field [36]. For these relatively simple proteins, the landscapes are not highly complex and generally exhibit a single funnel leading to the global minimum. The low-lying minima are structurally very similar and closely resemble the corresponding PDB structures, as anticipated. More complex behaviour emerges for the de novo designed protein (Fig. 4c), where the lowest-energy minima display the largest structural diversity and the associated funnel is notably elongated, a feature not typically observed in landscapes of natural proteins. This suggests that de novo designs may possess broader or less strongly biased folding landscapes, motivating future studies aimed at connecting protein design principles with folding trajectories. Additional deviations are observed for apo calbindin D9k (Fig. 4b), where low-lying minima lacking the $\beta$-hairpin are energetically favoured. This finding opens an interesting avenue for further investigation of alternative folding pathways leading to stable open conformations, potentially linked to the biological function of calbindin, which involves calcium binding and transitions between open and closed states.

Exploration of the energy landscapes of A$\beta _{28}$, A$\beta _{40}$, and A$\beta _{42}$ (Fig. 4e–g) reveals pronounced differences in landscape topology and structural heterogeneity. Both A$\beta _{28}$ and A$\beta _{42}$ exhibit multifunnel landscapes, characteristic of intrinsically disordered systems [21–23]. This ruggedness reflects the coexistence of multiple competing structural motifs and has been linked to transitions from predominantly $\alpha$-helical conformations to $\beta$-hairpin structures, a key step in amyloid aggregation and plaque formation [172]. Such frustrated landscapes lack a well-defined global minimum and are instead characterised by numerous low-lying minima separated by high free energy barriers. In contrast, A$\beta _{40}$ displays a comparatively simple, near single-funnel landscape dominated by compact, largely $\alpha$-helical states. While this may partly reflect undersampling or the presence of high barriers separating alternative basins, it nonetheless indicates a more restricted conformational ensemble within the explored landscape. Structural analysis further underscores these distinctions [21]. A$\beta _{28}$ samples a broad range of conformations, consistent with high flexibility and disorder, whereas A$\beta _{40}$ remains predominantly compact and occupies a narrower energetic window, suggesting enhanced stability within its principal basin. A$\beta _{42}$ occupies an intermediate regime, populating both compact and extended states, consistent with its increased aggregation propensity. These differences correlate with established size-dependent solubility trends in amyloid peptides, with A$\beta _{28}$ being the most soluble and A$\beta _{40}$ and A$\beta _{42}$ exhibiting lower solubilities. Collectively, these results highlight how subtle sequence variations reshape amyloid energy landscapes and aggregation behaviour.

Taken together, the UNRES energy landscapes highlight a clear distinction between simple globular proteins and amyloid peptides. Well-folded globular proteins are characterised by relatively smooth, single-funnel landscapes that guide folding efficiently towards a unique native state, with low-lying minima closely resembling experimental structures (Fig. 4a–d). In contrast, amyloid peptides populate rugged, multifunnel landscapes with numerous competing low-energy states separated by substantial barriers, reflecting intrinsic disorder and a strong sensitivity to sequence length and composition (Fig. 4e–g). These frustrated landscapes underpin the coexistence of diverse structural motifs and facilitate transitions associated with aggregation and fibril formation. Viewed through an energy landscape framework, UNRES provides a unified description that captures both the funnelled folding of stable proteins and the heterogeneous, aggregation-prone behaviour of amyloids, illustrating how landscape topology encodes fundamental differences in biomolecular function and misfolding.

## Conclusions

Energy landscape theory provides a powerful and unifying framework for understanding protein structure, dynamics, and function across theoretical resolution. Rather than static pictures, protein energy landscapes reveal scale-dependent representations generated by successive reductions of the underlying molecular Hamiltonian. From quantum mechanical PES to coarse-grained potentials of mean force, each level of description reshapes the landscape by smoothing microscopic roughness, renormalising barriers, and modifying basin stability, while often preserving the essential topological features that govern thermodynamics and kinetics. Well-folded globular proteins are characterised by robust, funnelled landscapes that remain qualitatively stable under coarse-graining, enabling reduced-resolution models to capture native-state organisation and dominant folding pathways with high reliability. Amyloidogenic peptides are presented by frustrated, multifunnel landscapes with numerous low-lying basins and high barriers, supporting heterogeneous conformational ensembles and multiple aggregation-prone pathways. These qualitative distinctions persist across modelling resolutions and are naturally rationalised within a landscape framework.

By combining atomistic force fields, coarse-grained models, and multiscale hybrid approaches within the Cambridge energy landscape framework, this review has illustrated how folding pathways, metastable states, and kinetic bottlenecks can be analysed consistently across scales. Complementary approaches for visualising energy landscapes, including MDDG and disconnectivity graphs, have also been discussed. In addition, we highlight methods that map biased MD simulations onto effective energy landscapes, notably Markov state models and enhanced-sampling techniques. Finally, commonly used structural descriptors such as the root-mean-square deviation and radius of gyration can be used to characterise conformational ensembles. Case studies ranging from BPTI and protein–glycosaminoglycan complexes to amyloid-$\beta$ peptides demonstrate that reduced-resolution models succeed not through detailed reproduction of microscopic interactions, but through preservation of basin connectivity, barrier hierarchies, and dominant transition pathways. Implicit solvent treatments, coarse-graining, and hybrid QM/MM schemes can therefore be understood as controlled projections of the underlying landscape that retain its essential organisation.

Taken together, these observations reinforce the view that landscape topology, rather than chemical detail alone, underpins the predictive power of protein modelling. Adopting a landscape-aware perspective allows biomolecular simulation to move beyond debates over accuracy versus efficiency towards a more nuanced understanding of resolution, projection, and robustness. As multiscale methods continue to develop, energy landscape theory will remain central to connecting molecular-level descriptions with emergent biological function and dysfunction.

## Data Availability

No datasets were generated or analysed during the current study.
